# A prognostic index model for predicting long-term recurrence of uterine leiomyoma after myomectomy

**DOI:** 10.1371/journal.pone.0254142

**Published:** 2021-07-01

**Authors:** Xiu Ming, Junying Zhou, Jinhai Gou, Na Li, Dan Nie, Luqi Xue, Zhengyu Li

**Affiliations:** 1 Department of Gynecology and Obstetrics, West China Second University Hospital, Sichuan University, Chengdu, People’s Republic of China; 2 Key Laboratory of Birth Defects and Related Diseases of Women and Children (Sichuan University), Ministry of Education, Chengdu, People’s Republic of China; 3 Gynecological Operative Room, West China Second University Hospital, Sichuan University, Chengdu, People’s Republic of China; 4 Sichuan Key Laboratory of Obstetrics & Gynecologic, West China Second University Hospital, Sichuan University, Chengdu, People’s Republic of China; 5 Department of Obstetrics and Gynecology, The First Affiliated Hospital of Zunyi Medical University, Zunyi, Guizhou, People’s Republic of China; 6 Department of Gynecology and Obstetrics, The Affiliated Hospital of Southwest Medical University, Luzhou, People’s Republic of China; Dipartimento di Scienze Mediche e Chirugiche (DIMEC), Orsola Hospital, ITALY

## Abstract

**Introduction:**

Uterine leiomyoma (UL) is a common benign pelvic tumor in women that has a high recurrence rate. Our aim is to propose a prognostic index (PI) model for predicting the long-term recurrence risk of uterine leiomyoma (UL).

**Methods:**

A total of 725 women who underwent myomectomy were enrolled in this retrospective multicenter study. Patients were contacted for follow-up. A PI model was proposed based on the multivariate Cox regression analysis in the model group. The predictive value of this model was tested in both internal and external validation group.

**Results:**

PI formula = 1.5(if 3–5 leiomyomas) or 2(if >5 leiomyomas)+1(if residue)+1(if not submucosal)+1(if combined endometriosis). The PI value was divided into low-risk, intermediate-risk, and high-risk group by cut-off values 1.25 and 3.75. In the model group, the high-risk group had a significantly 4.55 times greater recurrence risk of UL than that in the low-risk group [cumulative recurrence rate (CR): 82.1% vs 29.5%, HR = 4.55, 95% CI 2.821–7.339]; the intermediate-risk group had a significantly 2.81 times greater recurrence risk of UL than that in the low-risk group (CR: 62.3% vs 29.5%, HR = 2.81, 95% CI 2.035–3.878). The differences between any two risk groups were also significant (*P*< 0.05) in both internal and external validation groups.

**Conclusion:**

The model was proved to be effective in predicting recurrence of UL after myomectomy.

## Introduction

Uterine leiomyoma (UL) is the most common female pelvic benign tumor [1]. UL presents with clinical symptoms such as pelvic pain, menorrhagia, and pelvic mass, affecting an array of reproductive-aged women [2]. Hysterectomy has been proved as the most effective treatment for symptomatic UL [3]. However, it is not the preferred option for reproductive women who wish to preserve their uterus or fertility [4]. Therefore, myomectomy is widely used as an alternative with the potential for subsequent intervention [5]. Considering the high recurrence rates of 52.8%-57.7% at 60 months post laparoscopic myomectomy [6, 7] and 35.2%-47.0% post laparotomic myomectomy [6–8], we conducted this multicenter retrospective study to propose a prognostic index (PI) model for assessing the long-term (at least 5 years) recurrence risk of UL. In women aged 45 years and older, the recurrence and reoperation rates for UL after myomectomy were only 17.1% and 1.1%, respectively [9]. To improve the accuracy and practicability of our prediction model, we narrowed down our selection criteria to only reproductive women aged 18–44 years.

Many clinical factors are reported to be related to the rate of UL recurrence, such as the number of leiomyomas, age of the patient, size of the leading leiomyoma, and surgical approaches [10–12]. The presence of more than one leiomyoma and age 30–40 years at the time of myomectomy were reported as risk factors for UL recurrence with recurrence rates of 38.71% and 31.25%, respectively, at 60 months postoperatively [10]. The women who had given birth after myomectomy had a lower rate of reoperation owing to UL recurrence than those who had not given birth (7.8% vs 21.3%) [13]. However, controversial results have been reported across studies [8, 10, 11].

Nowadays gynecologists still assess individual UL recurrence risk based on their own experiences, resulting in subjective clinical decision making. The aim of this study was to propose an objective and quantitative prediction model to assess the risk of UL recurrence after myomectomy.

## Materials and methods

All patients were contacted by telephone to obtain verbal informed consent approved by the Institutional Ethics Committee of West China Second University Hospital of Sichuan University (number 063 in 2018). We included reproductive women aged 18 to 44 years who had initially undergone myomectomy at one of the three hospitals (West China Second Hospital of Sichuan university, The First Affiliated Hospital of Zunyi Medical University, and The Affiliated Hospital of Southwest Medical University) between April 2012 and October 2013. They were contacted over the telephone for follow-up between July 2018 and November 2018.

The inclusion criteria were as follows: (i) age 18–44 years; (ii) patients who were hospitalized for UL; (iii) no history of myomectomy. The exclusion criteria were as follows: (i) patients who could not be contacted by telephone or refused/unable to report their postoperative condition; (ii) the presence of any immune disease (rheumatoid arthritis, ankylosing spondylitis, systemic lupus erythematosus, or thrombocytopenic purpura) to exclude the influence of an immune system disorder; (iii) lack of regularly scheduled transvaginal ultrasonography examination (every 1 to 2 years) within the follow-up period; (iv) the detection of pathology reported non-leiomyoma; (v) hysterectomy performed for other gynecological diseases within the follow-up period; (vi) congenital uterine anomalies; (vii) malignant tumor; (viii)accidental death.

We retrospectively collected the following data: patients’ demographics (age at surgery, height and weight at the time of surgery, and pregnancy and delivery after surgery), associated pelvic diseases (endometriosis, adenomyosis, or adenomyoma and adnexal benign mass), characteristics of the leiomyomas (number of leiomyomas on transvaginal ultrasonography, maximum diameter of the leading leiomyoma, the uterine volume, and the leiomyoma subclassification by sonogram), surgical approaches, residue, recurrence, time and treatment of recurrence, and subsequent contraceptive methods.

The recurrence of UL was defined as a newly found leiomyoma larger than 1 cm as detected by transvaginal ultrasonography at six months or later after myomectomy [8, 9, 14]. The uterine volume was calculated using the formula for the volume of a prolate ellipsoid [15]. Residue of UL was defined as the removal of a lesser number of leiomyomas during myomectomy than that reported to be present according to the preoperative transvaginal ultrasonography; a gynecologist’s confirmation of failure to remove all the leiomyomas from the uterus during myomectomy; or leiomyomas reported in the initial postoperative follow-up (within three months) ultrasonography. The leiomyoma subclassification system from the International Federation of Gynecology and Obstetrics (FIGO) (Submucosal: FIGO 0, 1 and 2; Others: FIGO 3 to 8) was used to classify the type of leiomyoma [16]. Patients who presented with leiomyomas at more than one uterine localization were classified according to the leading myoma’s location. The associated pelvic diseases were defined as endometriosis, adenomyosis, adenomyoma, and adnexal benign mass detected by the pathology [12, 17].

Statistical analysis was performed using the SPSS Statistics software (Version 21.0; SPSS Inc., Chicago, IL, USA). The categorical data were expressed by frequency and percentage. Continuous data were converted to categorical data with the utilization of the ROC curve and the Youden’s index. The cut-off value of BMI was determined by the international conventions for BMI groupings [18].

In the model group, univariate analysis for cumulative recurrence rates (CR) was executed through Kaplan–Meier methods, and variables with a *P*-value<0.1 by the log–rank test were taken into multivariable Cox proportional hazards (PH) regression analysis (using the Forward: LR) to assess their association with recurrence. Before executing multivariate analysis, we took the following three steps to make sure that use of the Cox survival model was appropriate. Step 1: The significant variables (*P*<0.1) in the univariate analysis were tested by the log cumulative hazards plots graphically [19] and/or time covariate test to confirm the PH assumption. No obvious cross of Kaplan–Meier survival curves combined with parallel curves of log cumulative hazards plots indicates one variable is under PH assumption. The *P* value ≥ 0.05 in the time covariate test also indicates one variable is under PH assumption. Step 2: A graphical display of hazard ratios (HRs) was plotted to test whether one variable with more than two categorical groups had a log-linear association with the risk of recurrence [19]. The HRs gained from univariate Cox regression analyses were used on the ‘y’ axis, and the medians of each of the categorical groups were used on the horizontal (‘x’) axis. The leiomyoma number on transvaginal ultrasonography went through this step. Step 3: The significant variables were assessed for multicollinearity by collinearity diagnostics. A variance inflation factor (VIF) ≥10 and/or a tolerance <0.2 indicates a problem of multicollinearity [20]. Statistical significance was considered at P < 0.05 in multivariate Cox regression analysis.

A prognostic index (PI) model was proposed based on the β-coefficients in the results of multivariate Cox regression analysis. The concordance was described by the C-statistic [21]. The PI value was divided into low-risk group, intermediate-risk group, and high-risk group by two cut-off values. Then the differences among the three groups were tested by Kaplan–Meier analysis (using paired log-rank test) and univariate Cox regression analysis (using the Forward: LR) in model group and validation groups. Statistical significance was considered at *P* < 0.05.

## Results

After a median follow-up of 69 (range: 65–74) months, 725 patients were finally included in this study from an initial sample of 1214 patients. In the remaining 489 (40%) patients, 187 (15%) patients were excluded according to the exclusion criteria and 302 (25%) patients were lost to follow-up. Patients from West China Second Hospital of Sichuan university were divided into the model group (390 patients) and the internal validation group (172 patients); 163 patients from the other two hospitals were included in the external validation group (S1 and S2 Figs).

The cut-off value for the maximum diameter of leading leiomyoma was 4 cm and the uterine volume was 1140 cm^3^ (S1 Table). After failing to find a significant cut-off value of age by Youden’s index, inspired by a published article [10], we stratified the age as 31–40 years versus the others. In the univariate analyses of the model group, 10 variables resulted in *P* value<0.1 (Table 1) and all of them were graphically under PH assumption (S3–S12 Figs). As for the time covariate test, nine variables got a *P* value >0.05, except the postoperative pregnancy or delivery, which got a *P* value = 0.045 (Table 1). Considering its *P* value was just slightly below 0.05 and it was graphically under PH assumption, we decided to add it into multivariate Cox regression analyses. A graphical display of the hazard ratios (HRs) was plotted for leiomyoma number and it showed that leiomyoma number had a log-linear relationship with the risk of recurrence (S13 Fig). Both the tolerance and VIF did not indicate any considerable multicollinearity among the selected 10 variables (S2 Table).

**Table 1:** Univariate analyses of variables in the model group by the Kaplan–Meier method.

Variables	Number	Recurrence N	CR	P value
**Age at surgery**				0.072
** 18–30 and 41–44**	191	76	39.8%	
** 31–40**	199	99	49.7%	
**BMI**				0.175
** ≤25 kg/m2**	352	163	46.3%	
** >25 kg/m2**	38	14	36.8%	
**Leiomyoma number on TVS**				<0.001
** 1**	197	59	29.9%	
** 2**	58	26	44.8%	
** 3–5**	83	53	60.2%	
** >5**	52	40	76.9%	
**Maximum diameter of the leading leiomyoma**				0.023
** ≤4 cm**	106	37	34.9%	
** >4 cm**	284	138	48.6%	
**Uterine volume**				0.002
** ≤1140cm**^**3**^	274	110	40.1%	
** >1140 cm**^**3**^	116	65	56.0%	
**Leiomyoma subclassification**				<0.001
** Submucosal**	104	30	28.8%	
** Others**	286	145	50.7%	
**Surgical approaches**				0.016
** H or/and L**	137	49	35.8%	
** T**	253	126	49.8%	
**Residue**				<0.001
** No**	310	122	39.4%	
** Yes**	80	53	66.2%	
**Combined endometriosis**				0.008
** No**	355	153	43.1%	
** Yes**	35	22	62.9%	
**Adenomyosis or adenomyoma**				0.592
** No**	363	164	45.2%	
** Yes**	27	11	44.9%	
**Adnexal benign mass**				0.411
** No**	356	157	44.1%	
** Yes**	34	18	52.9%	
**Postoperative Pregnancy or Delivery**				0.041
** No**	301	145	48.2%	
** Yes**	89	30	33.7%	
**Postoperative GnRH-α**				0.069
** No**	365	158	43.3%	
** Yes**	25	15	60.0%	
**Oral contraceptive pills**				0.344
** No**	378	171	45.2%	
** Yes**	12	6	50.0%	

CR: cumulative recurrence rates; Age: at time of surgery; TVS: transvaginal ultrasonography; H: hysteroscopic myomectomy; L: laparoscopic myomectomy; T: transabdominal myomectomy; GnRH-α: gonadotropin-releasing hormone agonists.

The results of multivariate Cox regression analyses, reported leiomyoma number [leiomyoma number (N) = 2: HR = 1.321, 95% confidence interval (CI) 0.776–2.248, *P* = 0.304; N = 3–5: HR = 2.079, 95% CI 1.315–3.286, *P* = 0.002; N>5: HR = 2.941, 95% CI 1.800–4.805, *P*<0.001], residue (HR = 1.501, 95% CI 1.025–2.197, *P* = 0.037), leiomyoma subclassification (HR = 1.598, 95% CI 1.006–2.537, *P* = 0.047), and combined endometriosis (HR = 1.711, 95% CI 1.049–2.791, *P* = 0.032) were independent predictors of UL recurrence. One to two leiomyomas were regarded as one group according to the *P* = 0.304 (Table 2).

**Table 2:** Multivariate Cox regression analyses and the prognostic index (PI) formula based on the β-coefficients.

Variables	β-coefficient	HR	95.0% CI (Lower-Upper)	P value	Simplified coefficient
**Leiomyoma number**				<0.001	
**1**		reference			0
**2**	0.279	1.321	0.776–2.248	0.304	0
**3–5**	0.732	2.079	1.315–3.286	0.002	1.5
**>5**	1.079	2.941	1.800–4.805	<0.001	2
**Residue**					
**No**		reference			0
**Yes**	0.406	1.501	1.025–2.197	0.037	1
**Leiomyoma subclassification**					
**Submucosal**		reference			0
**Others**	0.469	1.598	1.006–2.537	0.047	1
**Combined endometriosis**					
**No**		reference			0
**Yes**	0.537	1.711	1.049–2.791	0.032	1
**PI formula = 1.5(if 3–5 leiomyomas) or 2(if >5 leiomyomas)+1(if residue)+1(if not submucosal)+1(if combined endometriosis)**					

HR: hazard ratio; 95% CI: 95% confidence interval.

To form the model based on the β-coefficients, we doubled the β-coefficients to simplified Figures and then proposed the prediction formula as follows:

$$\text{PI}=1.5\left(\text{if}\;3–5\;\text{leiomyomas}\right)\;\text{or}\;2(\text{if}>5\;\text{leiomyomas})+1\left(\text{if}\;\text{residue}\right)+1\left(\text{if}\;\text{not}\;\text{submucosal}\right)+1\left(\text{if}\;\text{combined}\;\text{endometriosis}\right).$$

**Fig 1:**
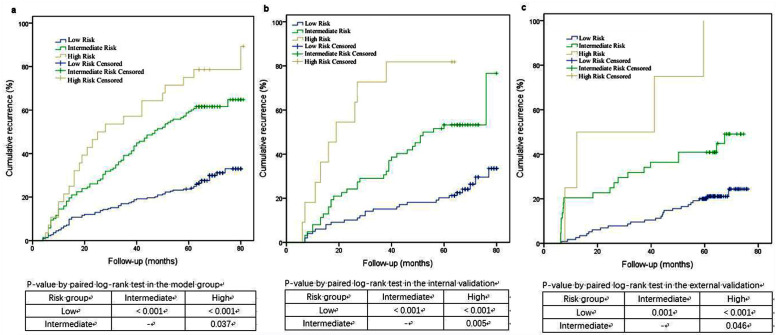
PI-based Kaplan–Meier analysis in (a) model group, (b) internal validation, and (c) external validation.

The C-index was 0.685 (95% CI 0.636–0.734) in the model group. The PI value (0–5.0) was divided into low-risk group, intermediate-risk group, and high-risk group by cut-off values 1.25 and 3.75 (Table 3). A higher PI value represented a higher recurrence risk. The recurrence differences between any two groups were all statistically significant (*P*< 0.05) in the model, internal validation, and external validation (Fig 1).

**Table 3:** PI-based Kaplan–Meier analysis and univariate Cox regression analysis in the model and validations.

Risk groups	N (%)	Recurrence	CR	HR	95% CI (Lower-Upper)
**Prediction model**	390 (100%)	175	44.9%		
**Low-risk**	224 (57.4%)	66	29.5%	reference	
**Intermediate-risk**	138 (35.4%)	86	62.3%	2.809	2.035–3.878
**High-risk**	28 (7.2%)	23	82.1%	4.550	2.821–7.339
**Internal validation**	172 (100%)	69	40.1%		
**Low-risk**	99 (57.6%)	26	26.3%	reference	
**Intermediate-risk**	62 (36.0%)	34	54.8%	2.805	1.668–4.716
**High-risk**	11 (6.4%)	9	81.8%	7.815	3.580–17.063
**External validation**	163 (100%)	49	30.1%		
**Low-risk**	115 (70.6%)	25	21.7%	reference	
**Intermediate-risk**	44 (26.9%)	20	45.5%	2.578	1.431–4.645
**High-risk**	4 (2.5%)	4	100%	8.724	3.002–25.357

CR: cumulative recurrence rates; HR: hazard ratio; 95% CI: 95% confidence interval.

In the model group, the high-risk group showed a significantly greater recurrence risk than the low-risk group (HR = 4.55, 95% CI 2.821–7.339, CR 82.1% vs 29.5%). The intermediate-risk group also showed a greater recurrence risk than the low-risk group (HR = 2.81, 95% CI 2.035–3.878, CR 62.3% vs 29.5%).

In the internal and external validation groups, the C-index were 0.703 (95% CI 0.632–0.774) and 0.704 (95% CI 0.622–0.785), respectively; the low-risk group showed the lowest risk of recurrence, which was 26.3% and 21.7%, respectively; the intermediate-risk group showed the intermediate risk of recurrence, which was 54.8% and 45.5%, respectively; and the high-risk group showed the highest risk of recurrence, which was 81.8% and 100%, respectively.

In total, the low-risk, intermediate-risk, and high-risk group respectively indicated 26.7%, 57.4%, and 83.7% recurrence rates of UL after myomectomy.

## Discussion

UL is known to have a high recurrence rate of 35.2%–52.8% after myomectomy [10, 12]. Presently, gynecologists assess the individuals’ UL recurrence risk based on their own experiences. An objective tool to assess the UL recurrence risk is essential. So, we proposed a model for assessing the risk of long-term UL recurrence in reproductive women, hoping to optimize the clinical decision making.

Four factors (leiomyoma number, residue, leiomyoma subclassification, and combined endometriosis) were independent prognostic factors. Leiomyoma number and residue have been reported to be the risk factors of UL recurrence in previous studies [9, 10, 14, 22]. The leiomyoma subclassification was not identified as a risk factor for UL recurrence in two studies [10, 22], but instead they grouped the leiomyoma subclassification as FIGO 2–6 vs FIGO 7 or intramural vs subserous vs pedunculated, which was different from our groups as submucosal vs others (FIGO 0–2 vs FIGO 3–8). One study reported that associated pelvic disease was a risk factor for UL recurrence and one of these pelvic diseases was endometriosis [12].

Postoperative pregnancy or delivery was not an independent prognostic factor in some previous studies [9, 10]. However, some other studies have reported it to be associated with a lower risk of recurrence [12, 22–24]. In our research, it was found to be statistically associated with a lower risk of recurrence in univariate analyses but not in multivariate analyses. We think this factor’s influence on UL recurrence may vary with its times, lasting period, etc. Therefore, it remains a controversial risk factor. Moreover, a systematic review reported a significant growth of UL during the first trimester of pregnancy, a slowdown during mid pregnancy and a size reduction during late pregnancy and puerperium. The overall modification of UL during pregnancy and puerperium remains unclear [25]. Age at surgery is also a controversial risk factor. Previous studies grouped age differently when exploring its relationship with UL recurrence [10, 12, 14, 22]. From our results, we concluded that it was not an independent prognostic factor for UL recurrence in women aged 18–44 years. We assume the reasons why the women aged 31–40 years resulted in higher UL recurrence rate postoperatively than the others in our univariable analyses are that younger women may give birth after myomectomy, and older women would develop ovarian function decreases. Both reasons are related to the sex hormones change, which are widely believed to contribute to the growth of UL [26].

Approximately 7% patients had reoperation during our follow-up. These patients may benefit more from the choice of hysterectomy at the initial treatment, especially when they do not need to preserve their fertility or the female organ’s morphologic integrity. This model provides an evidence-based tool to individually estimate the approximate rate of LU recurrence, so as to aid in the choice of surgical methods.

Although not all the recurrent fibroids will require reintervention, the model may have the potential to contribute to the subsequent therapy or medical advices. A prior study has reported the use of oral contraceptive pills postoperatively to be protective against UL recurrence [23]. Although this kind of findings has not been widely accepted, it provides the possibility that patients in the high-risk group of recurrence may benefit from subsequent therapy that could lower the risk of UL recurrence after myomectomy in the future. Recently, the benefits to remove asymptomatic submucous myomas in women of reproductive age are debated. These myomas may impact embryo implantation and pregnancy outcomes through anatomic modification of the endometrial cavity as well as alteration of the intrauterine microenvironment [27, 28]. So, the Global Congress on Hysteroscopy Scientific Committee recommends hysteroscopic myomectomy when asymptomatic submucous myomas ≥15 mm is found in women with immediate fertility request [29]. Giving birth earlier could be recommended to women who have birthing plan and result in high risk of UL recurrence after myomectomy on condition that the risk of uterine rupture is low enough. Some women would be anxious about UL recurrence. The estimated rates of UL recurrence by the model could release them if the risk is low.

The limitation is that the participants are East Asians. It needs to be investigated whether the model can be applied to the Caucasians, as well as the Black and Hispanic populations. Moreover, the Chinese population may have a lower parity rate, which could make our results not applicable to other Asian patient populations. In our study, all the hysteroscopic myomectomy was performed using the traditional hysteroscopic resectoscope with myoma extraction and without using hysteroscopic morcellator. Some studies are debating the new techniques like office hysteroscopic myomectomy without myoma extraction and Hysteroscopic Tissue Removal systems (HTRs) [30, 31]. These new techniques may impact the recurrence rate of submucosal myoma. Estrogen and progesterone are reported as promoters of UL growth [28]. It is a pity that our model fails to include the two hormones due to lack of relative data. In addition, this is a retrospective design research with selection bias. However, both internal and external validation results were similar to the model group and all the C-index indicated acceptable concordance. Therefore, it would be reasonable to acknowledge the legitimacy of our model.

The model proved to be useful in distinguishing low-risk (26.7%), intermediate-risk (57.4%), and high-risk (83.7%) groups for long-term recurrence of UL after myomectomy in reproductive women. It can be an objective tool providing the approximate rate of UL recurrence for the clinical decision making. Further research, especially prospective research, should be carried out to confirm the predictive ability of this model and its clinical usage.

## Supporting information

S1 Table Cut off values of the continuous variables based on Youden’s index. (DOCX)

S2 Table Test results for proportional hazards (PHs) assumption and multicollinearity. (DOCX)

S1 Fig Flow chart of internal patient selection and distribution. (TIF)

S2 Fig Flow chart of external patient selection. (TIF)

S3 Fig Age at surgery in the model group. (a) Kaplan–Meier survival curves. (b) Log cumulative hazards plot.
(TIF)

S4 Fig Leiomyoma number on transvaginal ultrasonography in the model group. (a) Kaplan–Meier survival curves. (b) Log cumulative hazards plot.
(TIF)

S5 Fig Maximum diameter of leading leiomyoma in the model group. (a) Kaplan–Meier survival curves. (b) Log cumulative hazards plot.
(TIF)

S6 Fig Volume of uterine in the model group. (a) Kaplan–Meier survival curves. (b) Log cumulative hazards plot.
(TIF)

S7 Fig Leiomyoma subclassification in the model group. (a) Kaplan–Meier survival curves. (b) Log cumulative hazards plot.
(TIF)

S8 Fig Surgical approaches in the model group. (a) Kaplan–Meier survival curves. (b) Log cumulative hazards plot.
(TIF)

S9 Fig Residue in the model group. (a) Kaplan–Meier survival curves. (b) Log cumulative hazards plot.
(TIF)

S10 Fig Combined endometriosis in the model group. (a) Kaplan–Meier survival curves. (b) Log cumulative hazards plot.
(TIF)

S11 Fig Postoperative GnRH-αin the model group. (a) Kaplan–Meier survival curves. (b) Log cumulative hazards plot.
(TIF)

S12 Fig Postoperative pregnancy or delivery in the model group. (a) Kaplan–Meier survival curves. (b) Log cumulative hazards plot.
(TIF)

S13 Fig A graphical display of hazard ratios (HRs) of leiomyoma number obtained by transvaginal ultrasonography (TVS). (TIF)

S1 File Dataset. (XLSX)
