# Supplementary material for: A prognostic index model for predicting long-term recurrence of uterine leiomyoma after myomectomy
Source: PLoS One. 2021 Jul 1;16(7):e0254142. doi: 10.1371/journal.pone.0254142 (PMC8248613; doi:10.1371/journal.pone.0254142)
Supplement: S1 Table — (DOCX) [file pone.0254142.s001.docx]

**S1 Table****.** **Cut off values of the continuous variables based on Youden's index.**

|  | **AUC** | **P value** | **Youden's index** | **Cut off value** |
| --- | --- | --- | --- | --- |
| **Volume of uterine** | 0.576 | 0.014 | 0.14 | 1140cm^3^ |
| **Maximum diameter of leading leiomyoma** | 0.560 | 0.050 | 0.135 | 4cm |

ROC: receiver operator characteristic curve; AUC: area under ROC curve.
