# Supplementary material for: A prognostic index model for predicting long-term recurrence of uterine leiomyoma after myomectomy
Source: PLoS One. 2021 Jul 1;16(7):e0254142. doi: 10.1371/journal.pone.0254142 (PMC8248613; doi:10.1371/journal.pone.0254142)
Supplement: S2 Table — (DOCX) [file pone.0254142.s002.docx]

**S2 Table.** **Test results for proportional hazards (PHs) assumption and multicollinearity**

| **Variables** | **Log cumulative hazards plots.** | **Time covariate test** | **Tolerance** | **VIF** |
| --- | --- | --- | --- | --- |
| **Age at surgery** | Yes | 0.355 | 0.963 | 1.038 |
| **Leiomyoma number on** **TVS** | Yes | 0.054 | 0.712 | 1.405 |
| **Maximum diameter of the leading leiomyoma** | Yes | 0.464 | 0.536 | 1.867 |
| **Volume of uterine** | Yes | 0.984 | 0.813 | 1.23 |
| **Leiomyoma subclassification** | Yes | 0.412 | 0.505 | 1.978 |
| **Surgical approaches** | Yes | 0.053 | 0.422 | 2.367 |
| **Residue** | Yes | 0.534 | 0.823 | 1.215 |
| **Combined endometriosis** | Yes | 0.841 | 0.886 | 1.129 |
| **Postoperative GnRH-α** | Yes | 0.063 | 0.893 | 1.12 |
| **Postoperative Pregnancy or Delivery** | Yes | 0.045 | 0.956 | 1.046 |

VIF: variance inflation factor; GnRH-α: gonadotropin-releasing hormone agonists; TVS: transvaginal ultrasonography.
