# Supplementary figures and images for: A prognostic index model for predicting long-term recurrence of uterine leiomyoma after myomectomy
Source: PLoS One. 2021 Jul 1;16(7):e0254142. doi: 10.1371/journal.pone.0254142 (PMC8248613; doi:10.1371/journal.pone.0254142)

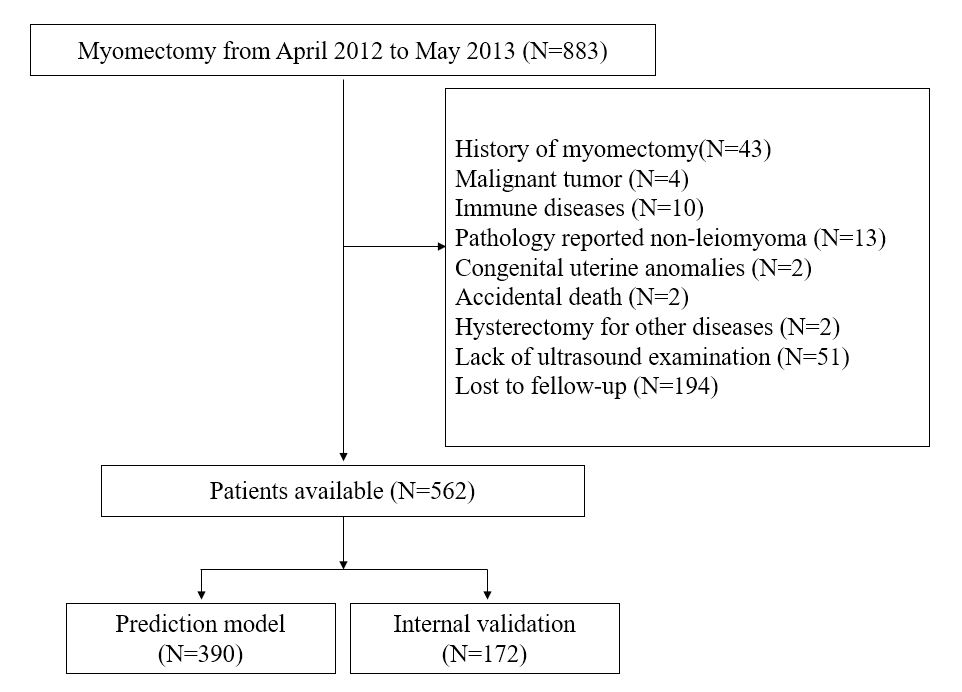

Supplement: S1 Fig — (TIF) [file pone.0254142.s003.tif]

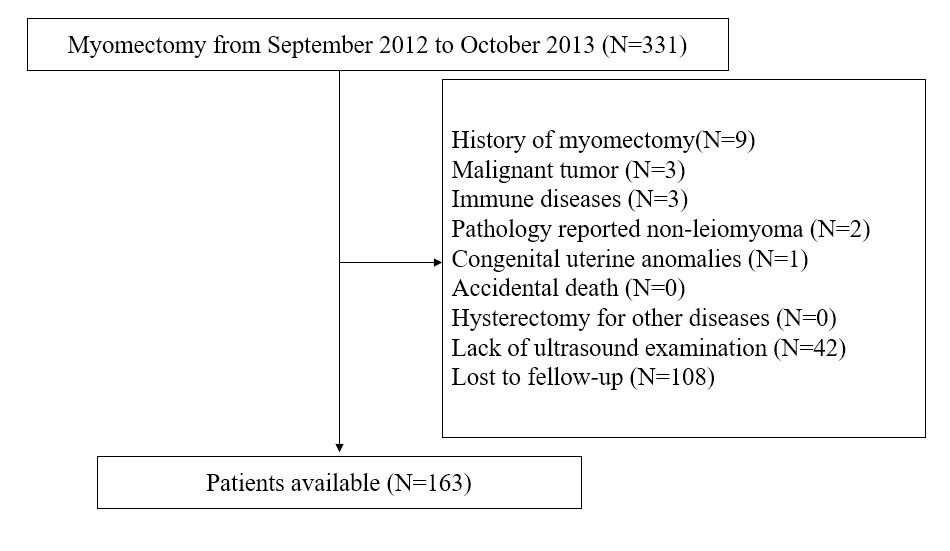

Supplement: S2 Fig — (TIF) [file pone.0254142.s004.tif]

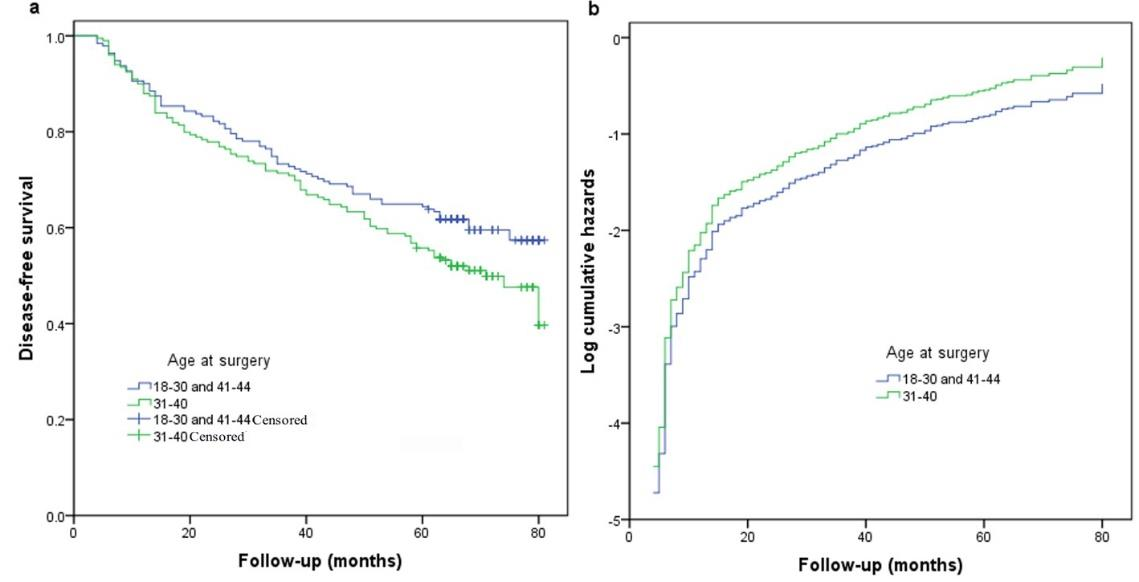

Supplement: S3 Fig — (a) Kaplan–Meier survival curves. (b) Log cumulative hazards plot. (TIF) [file pone.0254142.s005.tif]

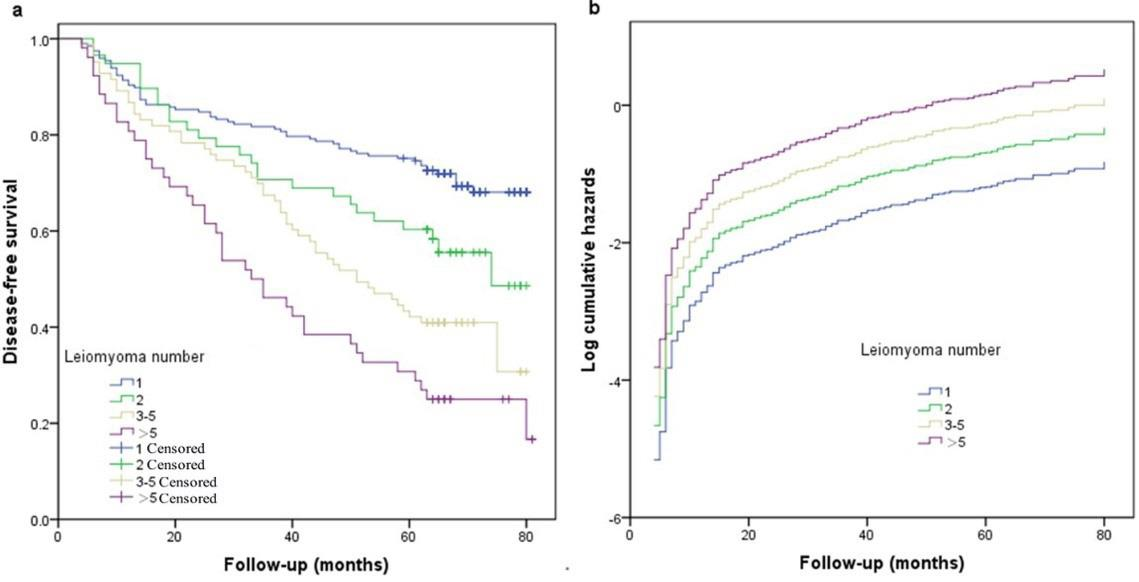

Supplement: S4 Fig — (a) Kaplan–Meier survival curves. (b) Log cumulative hazards plot. (TIF) [file pone.0254142.s006.tif]

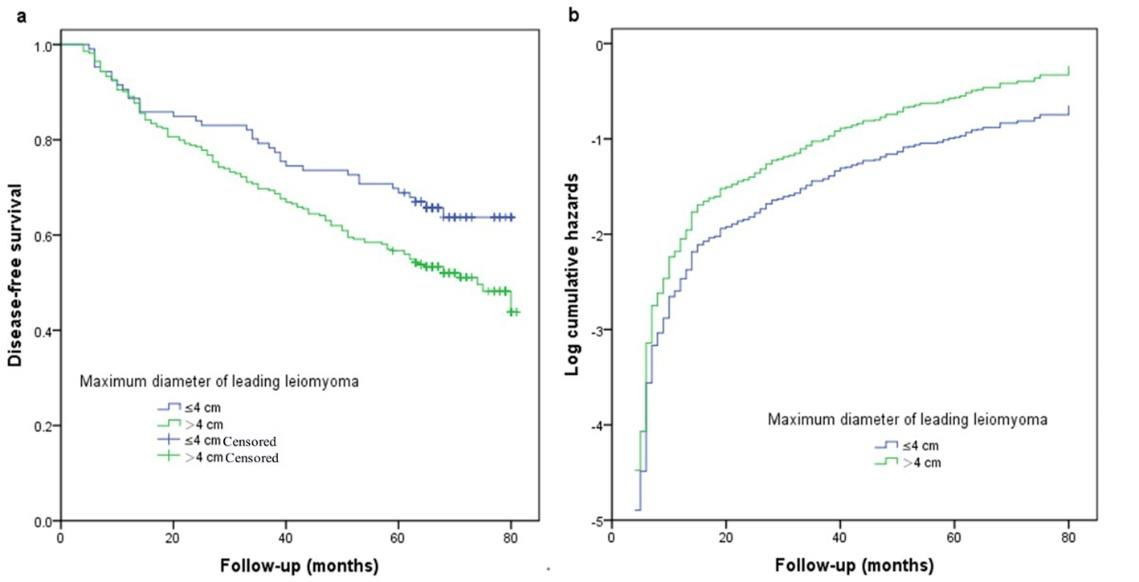

Supplement: S5 Fig — (a) Kaplan–Meier survival curves. (b) Log cumulative hazards plot. (TIF) [file pone.0254142.s007.tif]

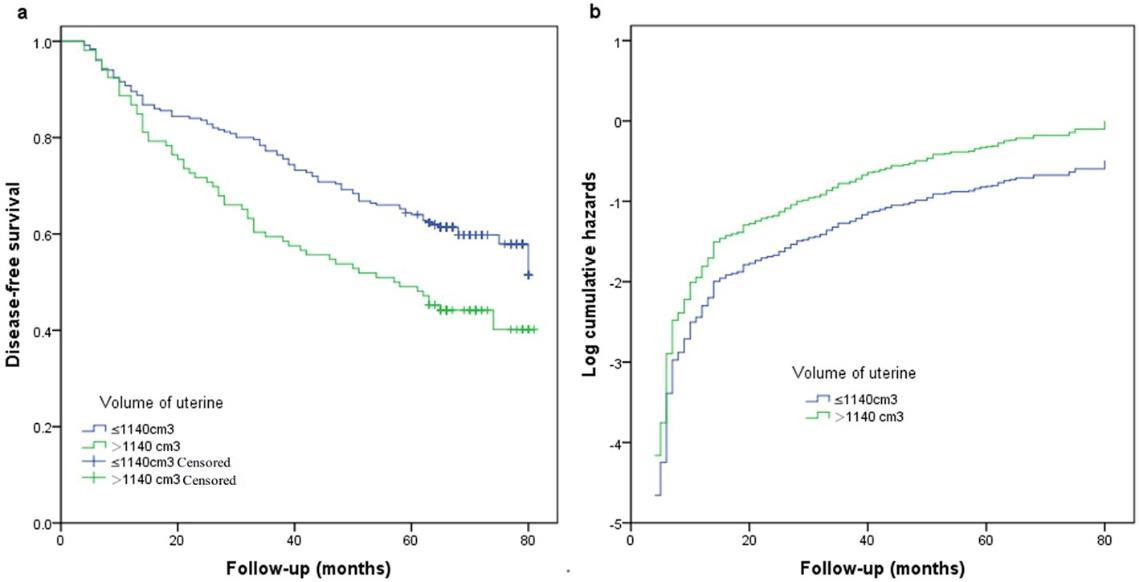

Supplement: S6 Fig — (a) Kaplan–Meier survival curves. (b) Log cumulative hazards plot. (TIF) [file pone.0254142.s008.tif]

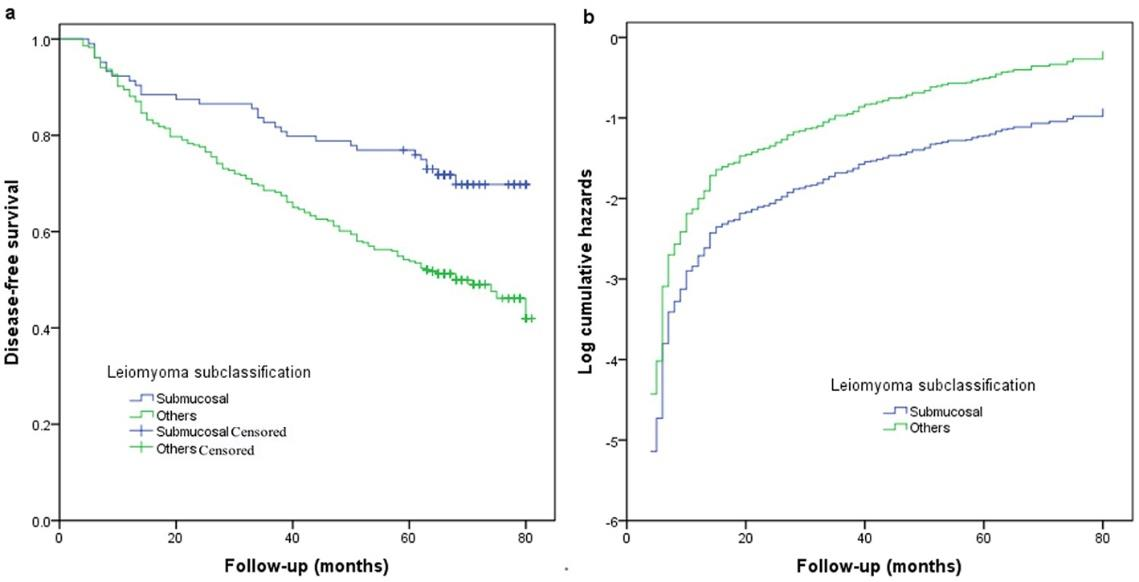

Supplement: S7 Fig — (a) Kaplan–Meier survival curves. (b) Log cumulative hazards plot. (TIF) [file pone.0254142.s009.tif]

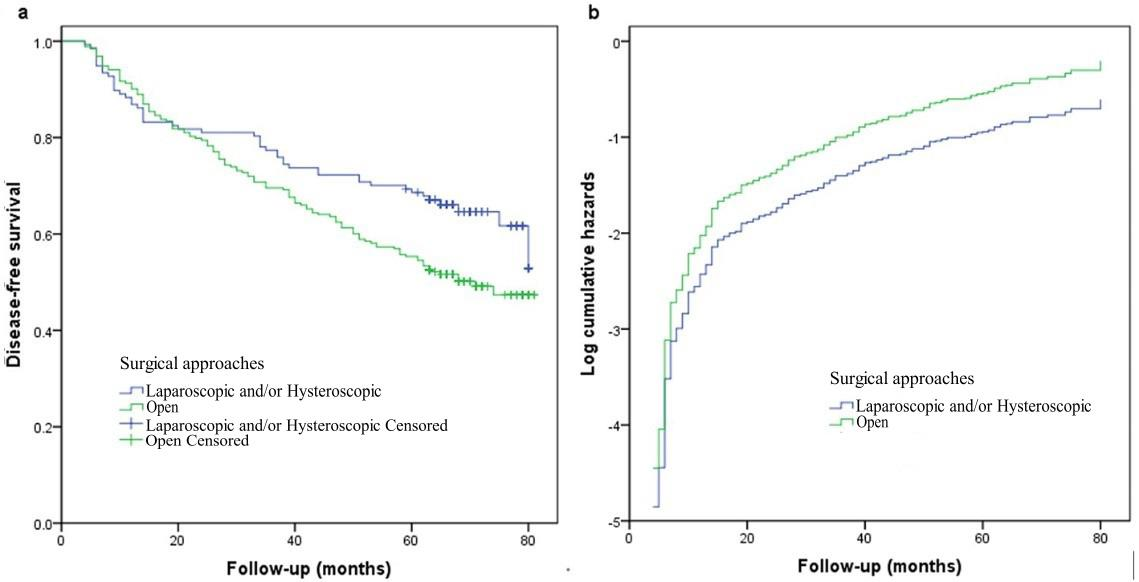

Supplement: S8 Fig — (a) Kaplan–Meier survival curves. (b) Log cumulative hazards plot. (TIF) [file pone.0254142.s010.tif]

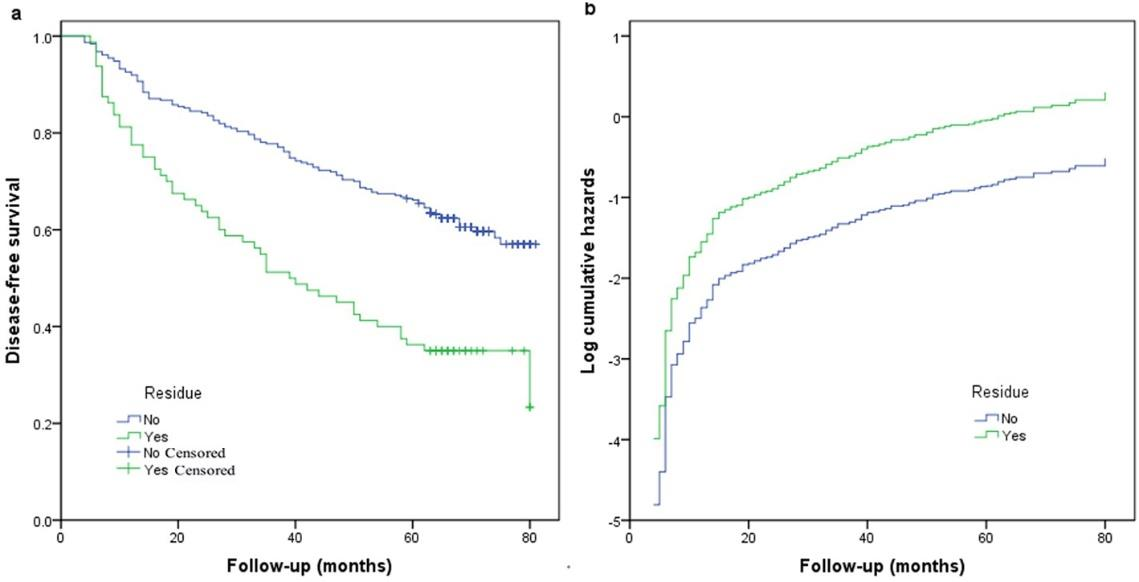

Supplement: S9 Fig — (a) Kaplan–Meier survival curves. (b) Log cumulative hazards plot. (TIF) [file pone.0254142.s011.tif]

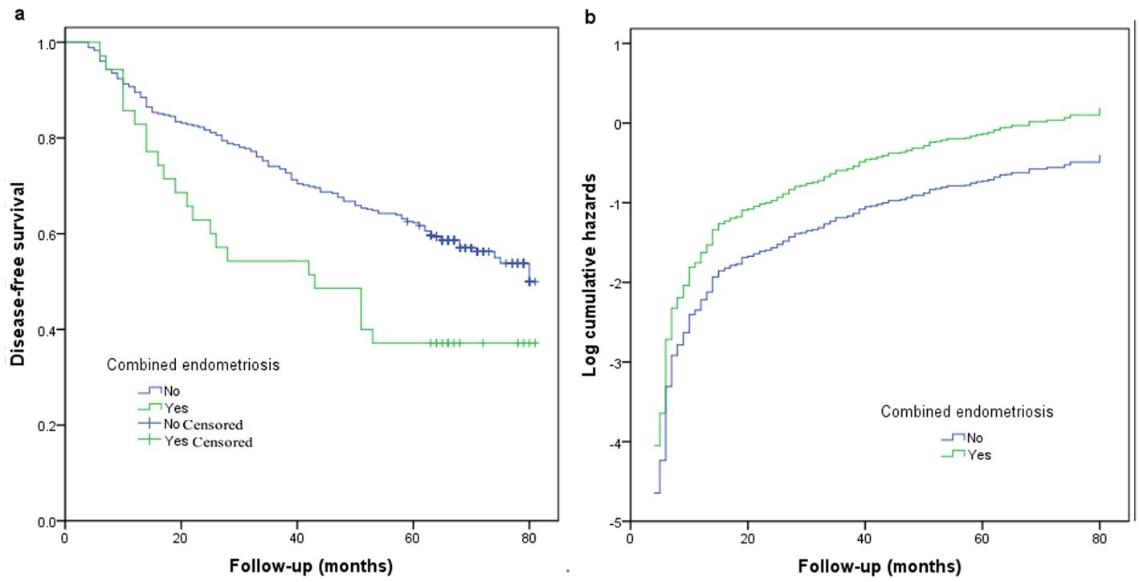

Supplement: S10 Fig — (a) Kaplan–Meier survival curves. (b) Log cumulative hazards plot. (TIF) [file pone.0254142.s012.tif]

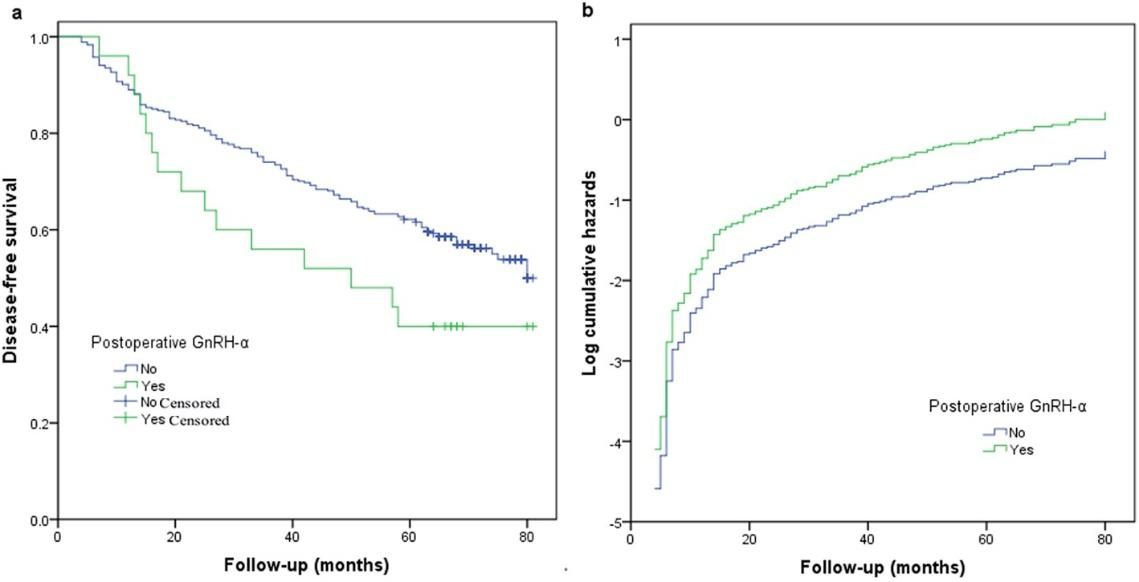

Supplement: S11 Fig — (a) Kaplan–Meier survival curves. (b) Log cumulative hazards plot. (TIF) [file pone.0254142.s013.tif]

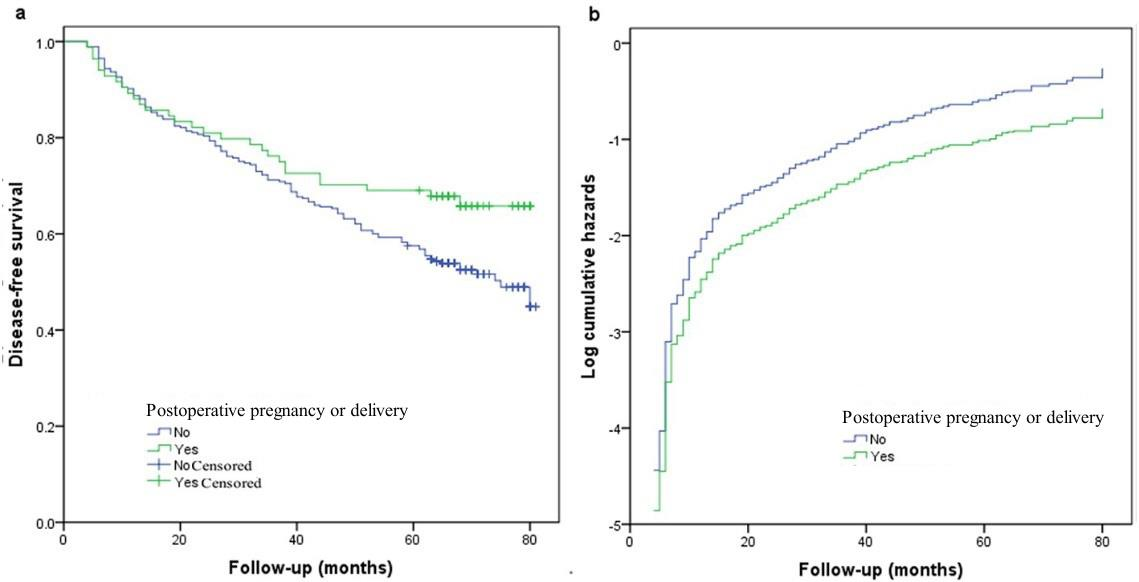

Supplement: S12 Fig — (a) Kaplan–Meier survival curves. (b) Log cumulative hazards plot. (TIF) [file pone.0254142.s014.tif]

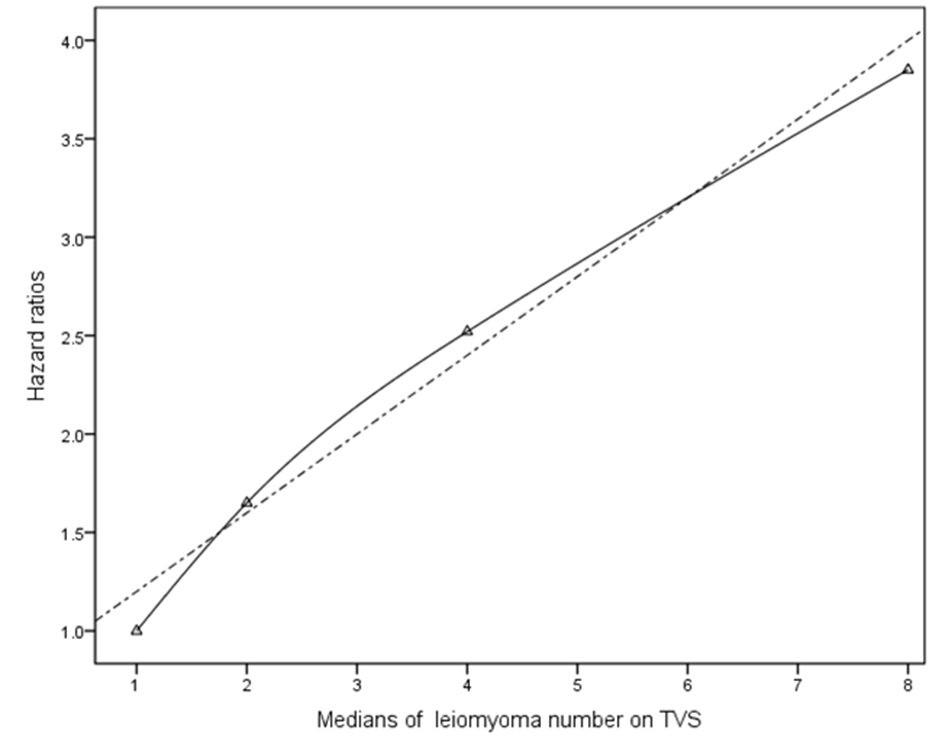

Supplement: S13 Fig — (TIF) [file pone.0254142.s015.tif]
